# Authorship ethics in global health research partnerships between researchers from low or middle income countries and high income countries

**DOI:** 10.1186/1472-6939-15-42

**Published:** 2014-05-28

**Authors:** Elise Smith, Matthew Hunt, Zubin Master

**Affiliations:** 1 Institut de Recherche en Santé Publique (IRSPUM), University of Montreal, Montreal, Canada; 2 Applied Social Sciences, Faculty of Arts and Sciences, University of Montreal, Montreal, Canada; 3 School of Physical and Occupational Therapy, McGill University, Montreal, Canada; 4 Centre for Interdisciplinary Research in Rehabilitation, Montreal, Canada; 5 Alden March Bioethics Institute, Albany Medical College, Albany, NY, USA; 6 Health Law Institute, University of Alberta, Edmonton, AB, Canada

**Keywords:** Authorship, Global health research, Global health research partnerships, Collaboration, Ranking of authorship

## Abstract

**Background:**

Over the past two decades, the promotion of collaborative partnerships involving researchers from low and middle income countries with those from high income countries has been a major development in global health research. Ideally, these partnerships would lead to more equitable collaboration including the sharing of research responsibilities and rewards. While collaborative partnership initiatives have shown promise and attracted growing interest, there has been little scholarly debate regarding the fair distribution of authorship credit within these partnerships.

**Discussion:**

In this paper, we identify four key authorship issues relevant to global health research and discuss their ethical and practical implications. First, we argue that authorship guidance may not adequately apply to global health research because it requires authors to write or substantially revise the manuscript. Since most journals of international reputation in global health are written in English, this would systematically and unjustly exclude non-English speaking researchers even if they have substantially contributed to the research project. Second, current guidance on authorship order does not address or mitigate unfair practices which can occur in global health research due to power differences between researchers from high and low-middle income countries. It also provides insufficient recognition of “technical tasks” such as local participant recruitment. Third, we consider the potential for real or perceived editorial bias in medical science journals in favour of prominent western researchers, and the risk of promoting misplaced credit and/or prestige authorship. Finally, we explore how diverse cultural practices and expectations regarding authorship may create conflict between researchers from low-middle and high income countries and contribute to unethical authorship practices. To effectively deal with these issues, we suggest: 1) undertaking further empirical and conceptual research regarding authorship in global health research; 2) raising awareness on authorship issues in global health research; and 3) developing specific standards of practice that reflect relevant considerations of authorship in global health research.

**Summary:**

Through review of the bioethics and global health literatures, and examination of guidance documents on ethical authorship, we identified a set of issues regarding authorship in collaborative partnerships between researchers from low-middle income countries and high income countries. We propose several recommendations to address these concerns.

## Background

There has been considerable coverage in the popular press and in academic scholarship about authorship ethics including such topics as plagiarism, ghost authorship, and even the selling of authorship [1-6]. Authorship is very important to researchers in all disciplines because it directly impacts decisions regarding hiring, tenure and promotion, and awards and grants. Notwithstanding the guidance on ethical authorship and publication practices, several reports show that unethical authorship occurs in upwards of 10% of cases [7,8]. Disagreement about authorship allocation also occurs frequently within research teams. In a recent study of Nigerian researchers, 36.4% of respondents reported authorship disagreements [9]. Similarly, an editorial published by Zachariah et al. describes difficulties associated with distributing authorship in research teams conducting operational research in low and middle-income countries (LMICs) [10]. These authors note that the application of guidelines on authorship often excludes recognition of important actors who have made essential contributions to research in LMICs (e.g., non-governmental organisations, policy makers). Fair distribution of authorship has also been a concern in global health research (GHR) partnerships [11,12], where scholars from LMICs collaborate with researchers from high-income countries (HICs).

It is generally accepted that authorship credit should be given to individuals who make “substantial contributions” to the design and/or conduct of research, and the reporting of research [13]. However, many other factors come into play during decision-making around authorship credit, including: disciplinary norms [14], competition [15], departmental politics [16], and favouritism [17]. Authorship practices in the context of GHR can be even more challenging given the variety of roles and responsibilities of researchers from LMICs and HICs. Ultimately, various factors may contribute to, or influence authorship decisions.

The aim of this paper is to explore issues relating to authorship practices in GHR partnerships and to propose recommendations. We limit our focus on partnerships in which authors from LMICs collaborate with researchers from HICs. We begin by providing important background information including the general context of global health research and authorship as well as current norms or guidelines concerning authorship. We then consider four specific issues related to authorship, namely: language barriers limiting opportunities for authorship attribution; the lack of guidance for ranking authors; the risk of gift authorship linked to perceptions of editorial bias for well-known researchers from English-speaking HICs; and, the impact of diverse cultural understandings and expectations regarding research contribution and ownership. The two former issues are linked to authorship shortcomings regarding authorship recommendations outlined in the International Committee of Medical Journal Editors (ICMJE) authorship guidance while the latter two concern authorship practices more generally. While some issues may appear to be practical in nature, we will demonstrate that they often have significant ethical consequences. To address the ethical and practical concerns about authorship in GHR partnerships, we recommend further research on norms and practices in GHR settings, additional training for researchers, and finally, improved guidance.

## Discussion

### The context of authorship in global health research

Global health “places a priority on improving health and achieving equity in health for all people worldwide” and “emphasizes transnational health issues, determinants and solutions.” ([18], p.1995). Research in the field of global health is often multi or interdisciplinary and is highly collaborative [18]. For the purposes of this paper, the focus will be on a specific subset of partnerships in global health: situations where LMIC and HIC researchers collaborate as members of multi- or interdisciplinary research teams, and where authorship is attributed to many individuals, each responsible for specific tasks. These collaborative partnerships bring together researchers from LMICs and HICs to share and maximize their diverse expertise, experiences, and perspectives within a knowledge network. For example, researchers from HICs and LMICs may obtain a grant to study the genetics of a tropical disease. HIC researchers may have scientific expertise and access to state-of-the-art technology required for the study. LMIC researchers from countries affected by this disease can provide scientific expertise, as well as valuable insight and knowledge respecting local realities and needs; they are also likely to have easier access to, and be more easily accepted by, research participants and local institutions than are “outsiders” [19]. Together they may be able to develop epidemiological genetic information that can lead to more effective public health interventions to treat the targeted disease.

Such partnership collaborations have been promoted, in part, to counter inequality and imbalance in GHR where researchers from HICs have not always studied topics of relevance to local communities in LMICs [20]. For GHR projects undertaken in LMICs, funders and research ethics boards often require that the research address local interests and needs. Accordingly, researchers involved in GHR partnerships are increasingly being held ethically responsible to support “community collaboration and engagement” [20].

While many researchers and commentators have acknowledged the laudable goals and benefits of such GHR partnerships, they have also pointed out practical challenges and limitations of such collaborations. These challenges include power inequities, communication barriers, diverging research priorities, as well as important differences in research cultures [21]. Even if there is little wide scale empirical evidence, the allocation of research credit through authorship is a key issue that is often raised [9-12]. It remains, however, unclear how authorship is or should be appropriately reflected through the order of attribution.

### Authorship policies in GHR

Various organisations such as the ICMJE [13], World Association of Medical Editors (WAME) [22] and the Committee on Publication Ethics (COPE) [23] have discussed ethical authorship and publication considerations in scientific manuscripts. However, health-based researchers and journals have not as yet consistently applied current guidance [24]. COPE mentions that editors should adopt guidance related to authorship, but does not propose specific criteria on authorship [23] and WAME provides very general guidance recommending that all authors make “substantial intellectual contribution” [22]. The ICMJE has set out more specific detailed authorship recommendations. ICMJE lists the following criteria for authorship:

1. Substantial contributions to the conception or design of the work; or the acquisition, analysis, or interpretation of data for the work; AND

2. Drafting the work or revising it critically for important intellectual content; AND

3. Final approval of the version to be published; AND

4. Agreement to be accountable for all aspects of the work in ensuring that questions related to the accuracy or integrity of any part of the work are appropriately investigated and resolved ([13], p.2).

While ICMJE authorship recommendations may at first glance seem onerous, they aim to ensure that all researchers who are authors are engaged throughout the life cycle of the research project including conducting the research, drafting, revision and approval of the article, and accepting responsibility for the work. While the ICMJE recommendations have been and are still at times the subject of criticism, they have become the leading standard in health science research, including GHR [24-26].

#### *Issues regarding authorship in GHR*

##### 

**Satisfying authorship criteria for researchers with limited English language abilities** Although the ICMJE recommendations are reflected in many scientific codes of ethics and have been adopted by many journals [24], they do not adequately address the more complex nature of research collaborations where contextual factors influence whether every researcher contributes to, or participates in, the *entire* research process. This is particularly true in GHR where language abilities may pose significant barriers.

In GHR partnerships, HIC researchers are more likely to have stronger English language abilities than their colleagues in LMICs. The latter may be at a distinct disadvantage in drafting part of or an entire article for English health sciences publications, or critically revising it for important intellectual content (ICMJE criterion 2). While it is possible to publish in scientific journals in languages other than English, these journals are less likely to be in international databases and would not receive similar international readership or exposure [27].

If LMIC researchers are unable to draft or critically revise the article, they would technically fail to satisfy the ICMJE recommendations for authorship. Thus a researcher who may have substantially contributed to the research by participating in the study’s conception or design, or the acquisition, analysis or interpretation of data (thereby satisfying condition 1), may not be named as author due to a lack of English reading and writing skills. Having a non-English speaking individual draft the article in their native language with the aid of a professional translator might prove burdensome because of the substantial translation costs, and would also necessitate the verification of the translated text by a native English speaker to ensure its accuracy. Consider as well, that most free online translation tools are fraught with errors, requiring massive edits by the English-speaking author or payments for additional editorial services. Due to financial costs and the added time required, many researchers may be reluctant to pursue such steps to satisfy ICMJE criteria.

The ICMJE recommendations clearly state that

[…] the criteria are not intended for use as a means to disqualify colleagues from authorship who otherwise meet authorship criteria by denying them the opportunity to meet criterion #s 2 or 3. Therefore, all individuals who meet the first criterion should have the opportunity to participate in the review, drafting, and final approval of the manuscript [13].

Providing adequate opportunity is a fundamental principle in the ethics of science and helps to prevent discrimination based on gender, race, and sexual orientation [28]. However, LMIC researchers may still fall short of satisfying all four ICMJE criteria even if ample opportunity is given to write, revise and approve the manuscript because of limited knowledge of the English language.

In a similar vein, all researchers who are authors need to be accountable for the work as indicated in ICMJE criterion 4, and to do this they need to satisfy criterion 3 and approve the final version of the text to be published. Researchers who cannot read or write in English might be able to partially satisfy criterion 3 using online translation tools. But similar to the point made above, translation generated by these tools is far from flawless and language discrepancies are likely to result in a loss in translation. While not a perfect approach, online translation tools will often be sufficient to allow researchers who cannot read in English to approve the final version of the article and be accountable for the published work.

However, even if ways are found to satisfy criteria 3 and 4, criterion 2 – drafting the work or revising it critically for important intellectual content – will be much more difficult for non-English writing researchers to satisfy for English publications. To limit the shortcoming of translation tools, the manuscript’s revision could be achieved through verbal discussions in order to ensure that *all* researchers agree that the manuscript properly conveys the research. The notion of “revising the article critically” (as stated in ICMJE criterion 3) could be interpreted differently in the GHR context to mean “reviewing” the article for important intellectual content so that there is less dependence on LMIC authors’ English writing abilities. In this sense, the LMIC author, like any other author, is still engaged in the drafting and review process of the manuscript and is still contributing in substantial ways to the research. Further examination is warranted as to how current ICMJE guidelines could contribute to systematic exclusion of some GHR researchers from authorship based on language ability. Ultimately, the aforementioned and other amendments to ICMJE should be given further consideration in order to avoid unfair exclusion of authors based on language.

##### 

**Ranking authors in GHR collaborations** The ICMJE recommendations state that authorship decisions should be made by all authors as a group, and that the corresponding author “should be prepared to explain the presence and order of these individuals” [29]. This does not suffice to address the complexities of GHR collaborations.

In the health sciences more generally, authorship is often attributed in descending order of contribution [3] although other methods may also be applied (e.g., alphabetical order, principal investigator/team leader being senior last author) making distribution quite variable [30]. Moreover, ranking would most likely not apply when a number of authors have contributed more or less equally. The comparative assessment of contributions may also be tricky for different types of work (e.g., technical tasks or conceptual contributions to the design of research). Since GHR collaborations are often interdisciplinary, other distribution methods from various disciplines may also be considered, including alphabetical order [31]. In large teams where ranking is complex because many have contributed relatively evenly, a mixed method of ordering may be applied: the first few authors are listed according to the importance of contribution in descending order, while others are named in alphabetical order. In the interest of accuracy and transparency, an asterisk may be added with a disclaimer mentioning that certain individuals have contributed equally. Akhabue and Lautenback’s empirical study on equal credit in top medical journals shows that disclaimers were a growing trend in medical journals from 2000 to 2007 [32]. Diversity in naming authors might be due to the fact that norms regarding ranking have never been formally codified.

The lack of guidance respecting authorship order in general can also be applied in GHR collaborations and could potentially create confusion and lead to insufficient recognition of LMIC researchers. For example, LMIC researchers may be given reduced ranking on the author byline because their contributions to subject recruitment, data collection, administration and analysis are categorized as “technical tasks” and may be considered of lesser value than drafting the manuscript. This reduced authorship ranking is ethically problematic because it may place LMIC researchers at a disadvantage, which could negatively affect their career prospects, access to research funds, and the scholarly recognition they deserve.

Unfairness stemming from a lack of clear ranking methods may also be the effect of power differentials within and between teams. HIC researchers are often better positioned to obtain the funding essential to GHR projects. Funding eligibility for the team may be deemed more valuable in securing resources. Consequently, individuals from LMICs with less funding leverage might accept, or feel obliged to accept, a lower ranking of authorship, even when their contribution is more substantive and deserving of higher authorship ranking. Power differentials often linked to research financing is certainly an issue in many contexts of research [33]. However, in partnerships between HIC and LMIC researchers, power differential are likely more pronounced because of greater discrepancies in access to research funds.

In order to address differences in types of contributions and mitigate disadvantages to LMIC researchers, some research teams have elected to alternate the order of authors based on geographic origin instead of maintaining the extent of contribution as the sole determining factor in ranking. For example, in an HIV study in Guatemala, the authors elected to alternate their names based on their geographic location (e.g., one author from Canada, one from Guatemala, etc.) [34]. The intent is to recognize the amount of contribution of each author, as well as to address the reality that the contributions of LMIC contributors might, by their very nature, receive lower authorship recognition than those made by HIC contributors. While seeking to address issues of equity in GHR partnerships, concerns about fairness would arise especially where levels and the types of contribution differed extensively.

In an attempt to level the playing field, HIC researchers may provide greater visibility and opportunity to researchers in LMICs by offering authorship or a higher ranking than was deserved based on contribution alone. The rationale behind this may be similar to that found in affirmative action or redistributive justice programs which seek to compensate for past and/or present discrimination or bias. It may indeed put LMIC researchers on a more equal footing with HIC researchers. However, it is far from clear that authorship – which should establish responsibility and accountability for research [35] – is the appropriate method to level this playing field. Building research capacity in LMICs, providing more opportunities for LMIC researchers to contribute to research, and expanding the possibility of LMIC researchers to secure grant funding, are more promising means to bring balance than undeserved authorship.

The use of a disclosure statement to explain authorship order can be effective in clarifying and justifying authorship order, or to avoid misinterpretations. Disclosure statements (or “contributorship”) usually outline the contribution of individuals in research design, data collection and analysis, and drafting; however, they are limited in that such declarations do not clearly indicate how authors were ranked based on the amount of work or its level of difficulty [35]. Regardless of such shortcomings, declaring author contributions has increased transparency regarding the type of work of each individual. Certainly, further discussion about effective strategies for authorship ordering in GHR is needed in order to establish fairness and opportunity for all team collaborators.

##### 

**Perceived editorial bias may promote unethical authorship practices** The underrepresentation of research from LMICs in peer-reviewed journals is well documented [36-39]. There are a number of reasons to explain this situation, such as the lack of infrastructure; inadequate human and financial resources in LMICs [40]; weaknesses in manuscript preparation (presentation, logic, language); and limited access to scientific literature [37]. Several studies suggest that there may also be an inherent bias by journal editors who favour research from English speaking researchers [4,41-44]. However, when discussing the potential bias in favour of well-known or “star” authors, Godlee and Dickersin report that “the available evidence is patchy […] and inconclusive” [45], p.96.

Regardless of whether editorial bias regarding well-known authors is widespread or not, certain researchers might *believe* in the existence of such bias. This perception could incentivise researchers from LMICs to bestow “prestige authorship” upon their collaborators from HICs by offering them senior author or primary (first) author positions. Researchers may think that this helps their chances of getting the paper accepted for publication. Researchers from HICs could use the same rationale and advocate that they should be given primary or senior author positions, arguing that it will increase the likelihood of having the paper accepted in a high impact journal.

Several strategies have been implemented to address the underrepresentation of research from LMICs. Journals provide editing services for non-English authors [46,47]; space is devoted to local research on issues relevant to specific regions; certain costs are subsidized for LMIC researchers and institutions [48]; and open access journals provide waivers for authors from LMICs [49]. While these initiatives are laudable, they do not eliminate perceptions of editorial bias for prominent well-known, English speaking researchers. Researchers from LMICs may still decide to provide prestige authorship to their HIC counterparts irrespective of measures to increase access and reduce costs associated with publication. It is necessary to look for other means to address this potential bias. Perhaps open discussion of this topic to reduce misconceptions may help avoid such issues. It might also be warranted to ask why such perceptions may be present and to assess empirically the extent to which such perceptions actually influence authorship practices.

##### 

**Differing values and practices related to research integrity** Most research on the responsible conduct of research (RCR) comes from HICs – more specifically the US – and only recently are we learning about RCR practices in LMICs [50]. Consequently, the values, practices and principles defining research integrity mainly reflect the social norms and values in HICs, and do not always adequately take into consideration the norms and values of LMIC researchers. There is also little research documenting cultural differences and the related perceptions and norms of research integrity across different countries and regions for both HICs and LMICs. Cultural differences between GHR collaborators are likely to play an important role in authorship decisions and may explain some authorship practices that fall outside ICMJE recommendations. Cultural differences can lead to conflicting views or positions regarding RCR. In some cultural contexts, it may be deemed appropriate or even required to give authorship credit out of respect (e.g., to senior colleagues, or directors of institutes). Of course, this runs counter to norms and practices endorsed by guidance documents like the ICMJE.

Gender bias might also lead to unethical authorship practices in GHR collaborative partnerships. In some settings, women may be less likely to be named authors in research publications as often as men even if they have made the same contributions to the project. For example, one study found that black women in South Africa are particularly marginalised and rarely given first author positions in collaborative publications [51]. While the rate of women conducting research and publishing in South Africa has increased during the last 20 years [52], there remains significant underrepresentation of women in scientific authorship. More knowledge is needed to truly understand the effect of gender bias on authorship distribution in GHR.

As GHR is often undertaken by multi/interdisciplinary, international teams comprised of researchers from a variety of backgrounds and cultures, there is likely to be divergence respecting authorship practices and expectations. These differences may lead to conflicts within GHR teams or questionable authorship practices, which in turn may jeopardize future collaborations, or tarnish reputations and careers. Global health researchers especially need to be mindful of these differences. Disputes over authorship in GHR collaborative teams can be minimized and may be handled more collegially if they are discussed openly at the outset of a project [53]. Increased awareness of cultural differences among GHR team members is important in order to establish a reciprocal understanding of the views of researchers from different cultures, and facilitate negotiations and buy-in with respect to authorship decisions.

#### *Recommendations for ethical authorship in GHR*

We recommend three initiatives to begin addressing authorship issues related to GHR collaborative partnerships: (1) to undertake research on research integrity (RRI) with a focus on authorship in GHR, (2) to increase awareness and understanding in the research community about authorship issues in the GHR context, and (3) to strengthen ethical guidance on authorship in GHR.

### Research on authorship norms and practices in GHR settings

Performing conceptual as well as empirical RRI is a necessary preliminary undertaking to understand the norms, values, and practices of authorship specific to GHR. RRI will address descriptive questions such as how authorship is allocated, how authors are ranked, and what factors influence authorship decisions. Policy related questions would include the following: Are current authorship standards sufficient? Do we need to reform authorship criteria? What should be the basis for authorship ordering in GHR? Description of GHR practices might help us understand how authorship is distributed and how researchers perceive important topics related to authorship such as responsibility and merit.

### Educating researchers about authorship in the GHR context

Raising awareness about ethical authorship can be done through RCR education. Given the relatively high prevalence of authorship issues and disputes [7-9], and the likelihood that researchers will face authorship issues early in their careers, training on authorship ethics should be included in graduate studies. This would introduce students to a culture of research integrity at the outset of their research careers and help them to better recognize problematic issues and understand the implications of unethical conduct. Training can lead to better understanding, negotiation and acceptance regarding authorship at the outset of a project to prevent or mitigate reduced morale and conflict about authorship. More specifically, in practice, researchers may learn a variety of ethical ways to distribute authorship in GHR by providing opportunity for contributors to become authors without creating systematic disadvantages or unfairness.

Education programs used to promote RCR vary considerably between countries [50,54]. While RCR training is obligatory in the U.S. for all NIH funded researchers [55,56], it is not implemented systematically in all HICs [54]. We have also not identified any systematic education programs in LMICs. However, more empirical research is necessary to document ongoing initiatives that have not yet been reported in the literature. While there is a general trend toward greater international cooperation and harmonization respecting RCR [57], there is little information concerning authorship in the context of GHR partnerships in educational material. This gap may be related to the limited knowledge about specific authorship practices in GHR partnerships, as well as sparse policy guidance. Therefore, in addition to educating researchers about authorship generally, there is a pressing need for training that addresses authorship issues in GHR partnerships. Course curricula should be inclusive and relevant to both HIC researchers and LMIC researchers who participate in GHR partnerships. This approach is consistent with the notion of collaborative partnership.

### Strengthening guidance on authorship in GHR settings

A growing number of health science journals have adopted the ICMJE recommendations in their policies [24,58]. The ICMJE recommendations help bring clarity, and provide useful reference points for the development and enhancement of authorship practices. However, it should be noted that GHR researchers are not primarily focused on trying to remedy broad or systemic inequities associated with authorship allocation. Rather, they are focused on conducting good science, answering relevant research questions, and advancing their careers within established norms and practices.

Despite the worthy efforts of the ICMJE, there may be circumstances in which global health researchers reasonably deserve credit for their contributions, but do not satisfy all of the authorship criteria; as well, they may be exposed to unethical authorship practices i.e., prestige or gift authorship that is influenced by the GHR context. In an attempt to acknowledge various researchers in GHR, some may opt to follow ICMJE and simply list other major contributors in the acknowledgement section. Yet this may be considered ethically problematic to the extent that these practices reflect a systematic disadvantage of LMIC researchers. Perhaps the necessity of “critically revising” or drafting a paper is simply not always realistic in the case of GHR. Alternatively, it may be appropriate to give authorship credit for critically reviewing the paper and ensure accountability through signed attestations of one’s contribution (e.g., data collection, analysis, writing and review).

As stated previously, authorship policies that address GHR collaborations should be founded on empirical data and conceptual research of authorship norms, values and practices. Expanding authorship policies to be more responsive to the GHR context is important for GHR researchers from both HICs and LMICs. This policy base is essential in guiding the development of best practices to address or counter disproportionate advantage due to geographic location, language ability, academic affiliation, institutional reputation, and access to resources in GHR collaborations. Best practices are not meant to be restrictive; quite the contrary, they serve to pre-empt potential issues and help ensure consistency in the ethical practice of authorship assignment and ranking. Many key actors should be involved in this important discussion regarding authorship guidance including researchers, funding agencies, scientific societies, and journal editors.

## Summary

In this paper, we report and discuss potential ethical issues related to authorship attribution in GHR. Despite the growth of GHR collaborative partnerships, very little has yet been done to understand the ethical underpinnings of fair authorship practices in this setting. Deficiency in authorship policies, perceptions of bias, and cultural differences are likely to contribute to questionable or unethical authorship practices in GHR settings. To address these issues and promote ethical authorship practices, several strategies should be pursued. Further conceptual and empirical research is needed to better understand authorship practices in GHR. Responsive and applicable authorship policies for GHR should be developed. Finally, training should be implemented to share knowledge and expose researchers to authorship norms and practices in GHR. If authorship issues are not dealt with, they may not only undermine the integrity of research, demoralize researchers, and damage important future GHR collaborations.

## Competing interest

The authors declare that they have no competing interests.

## Authors’ contributions

All authors developed the main ideas of this manuscript together. ES initially drafted the manuscript. MH and ZM critically revised the draft. All authors approved the final paper.

## Pre-publication history

The pre-publication history for this paper can be accessed here:

http://www.biomedcentral.com/1472-6939/15/42/prepub
